# Ankle Motion Is Associated With Soft Tissue Displacement in the Dorsal Thigh: An *in vivo* Investigation Suggesting Myofascial Force Transmission Across the Knee Joint

**DOI:** 10.3389/fphys.2020.00180

**Published:** 2020-03-06

**Authors:** Jan Wilke, Heloise Debelle, Sarah Tenberg, Andrew Dilley, Constantinos Maganaris

**Affiliations:** ^1^Department of Sports Medicine, Goethe University Frankfurt, Frankfurt am Main, Germany; ^2^School of Sport and Exercise Sciences, Liverpool John Moores University, Liverpool, United Kingdom; ^3^Department of Neuroscience, University of Sussex, Brighton, United Kingdom

**Keywords:** myofascial force transmission, ultrasound, range of motion, fascia, myofascial chains

## Abstract

Experiments in cadavers have demonstrated significant mechanical interactions between constituents of myofascial chains. However, evidence for such force transmission effects is scarce under *in vivo* conditions. The purpose of this trial was to examine the impact of ankle motion on soft tissue displacement of the dorsal thigh. Eleven healthy active individuals (26.8 ± 4.3 years, six males), in prone position and with the knee extended, underwent passive calf stretches (ankle dorsal extension) imposed by an isokinetic dynamometer. High-resolution ultrasound was used to simultaneously capture the displacement of the semimembranosus muscle, which was quantified by means of cross-correlation analysis. Inactivity of the leg muscles was controlled using surface electromyography (EMG). One participant had to be excluded due to major EMG activity during the experiment. According to a one-sample *t* test testing the difference to the neutral zero position, ankle dorsal extension induced substantial caudal muscle displacements (5.76 ± 2.67 mm, *p* < 0.0001). Correlation analysis (Spearman), furthermore, revealed a strong association between maximal dorsal extension and semimembranosus motion (rho = 0.76, *p* = 0.02). In conclusion, the present trial provides initial *in vivo* evidence for a mechanical force transmission between serially connected skeletal muscles. This means that local alterations of the mechanical tissue properties may modify flexibility in neighboring (superior or inferior) joints.

## Introduction

Fascia, the collagenous connective tissue surrounding the skeletal muscles, has long been regarded as a passive packing organ with limited significance for the locomotor system (van der Wal, 2009). However, recent research unveiled a far more complex role. Firstly, histological studies have demonstrated the intrafascial existence of myofibroblasts (e.g., in the gastrocnemius fascia; Bhattacharya et al., 2010). Their contraction, most probably mediated by the vegetative nervous system, can produce substantial increases of tissue stiffness in the long-term (Schleip et al., 2019). Secondly, already within minutes or hours, alterations of the water content, e.g., induced by isometric stretching, have been shown to significantly impact viscoelastic tissue properties (Schleip et al., 2012). The capacity of fascia to soften or harden in response to mechanical stimuli may be of particular importance due to another newly discovered feature. In contrast to prior assumptions, the surrounding fasciae do not separate, but connect the skeletal muscles. This architecture can be found between synergists and antagonists located parallel to each other (Yucesoy, 2010) as well as between muscles arranged in-series (Wilke et al., 2016a; Wilke and Krause, 2019).

As a consequence of intermuscular continuity (Yucesoy, 2010; Wilke et al., 2016a; Wilke and Krause, 2019) it has been hypothesized that the modification of local tissue properties can affect adjacent structures (Wilke et al., 2019a): if the linkages connecting two muscles are stiff enough, they may transmit force. In fact, experimental trials made such observation, showing that length changes of lower leg muscles induced mechanical strains in neighboring synergists and antagonists (Huijing and Baan, 2001; Huijing et al., 2011). Since removing the fibrous linkages between the muscles reduced this effect (Huijing and Baan, 2001), the previously produced strains seem to stem from a mechanical force transmission (Yucesoy, 2010). In their systematic review of cadaveric studies, Krause et al. (2016) aimed to answer the question as to whether mutual interactions would also occur between serially connected muscles. According to their findings, substantial forces can be transmitted, particularly in the posterior myofascial chain (plantar aponeurosis, gastrocnemius, hamstring muscles, lumbar fascia/erector spinae muscle). For instance, traction applied to the biceps femoris leads to a force transmission to the lumbar fascia (Vleeming et al., 1995). However, as cadavers (a) frequently exhibit alterations of mechanical tissue properties (e.g., due to fixation in solutions like formalin) and (b) do not produce neuromuscular activity, the findings of most studies investigating in-series mechanical force transmission cannot be extrapolated to *in vivo* conditions.

As indicated, to date, only few trials have examined the relevance of myofascial chains in the living organism. A couple of studies demonstrated remote flexibility increases following local exercise treatments (Grieve et al., 2015; Wilke et al., 2016b, 2017, 2019b; Joshi et al., 2018). These findings are intriguing, seemingly verifying the observations made *in vitro*. It seems plausible that a decrease in tissue stiffness, induced by the local interventions, can be transmitted to more cranial structures (e.g., from Hamstrings and to the neck muscles). However, as the measured outcome (range of motion) represents a functional parameter, the registration of non-local exercise effects does not provide a definite proof for myofascial force transmission under living conditions. The use of high-resolution ultrasound imaging can resolve this research deficit as it is able to visualize a non-stretched tissue during elongation of a neighboring, connected structure: If substantial forces would be transmitted through the linkage, a visible displacement of the non-stretched tissue should occur.

Using a simple experimental approach (Cruz-Montecinos et al., 2015) showed that an anterior pelvic tilt leads to a recognizable displacement of the gastrocnemius fascia. Their sonographic examination provides first indications for a cranial-caudal force transmission effect originating at the hip joint. However, in many movements of daily life and sports (e.g., walking, sprinting, jumping, squatting), forces are generated in the legs and hypothetically transmitted in direction of the trunk. In addition to this, all above described available *in vivo* trials examining the functional relevance of myofascial chains were (1) based on caudal-cranial force transmission and (2) mainly focused treatments around the ankle joint (plantar fascia massage, calf stretching). The present proof-of-principle study, therefore, aimed to elucidate for the first time, if a dorsal extension movement (also often referred to as dorsal flexion) of the ankle leads to a myofascial force transmission to the posterior thigh under *in vivo* conditions.

## Materials and Methods

### Ethical Standard

The experimental ultrasound study was approved by the local ethics committee and conducted according to the Declaration of Helsinki as well as the guidelines of Good Clinical Practice. All enrolled participants provided written informed consent.

### Sample

Eleven healthy active individuals (26.8 ± 4.3 years, 6♂, 5♀) volunteered to participate. Exclusion criteria included severe orthopedic, cardiovascular, neurological, endocrine and psychiatric diseases, acute inflammation or history of surgery in the lower limb, intake of drugs that modify pain perception and proprioception, muscle soreness and pregnancy or nursing period. Recruitment was performed by word of mouth.

### Experimental Approach

**FIGURE 1:**
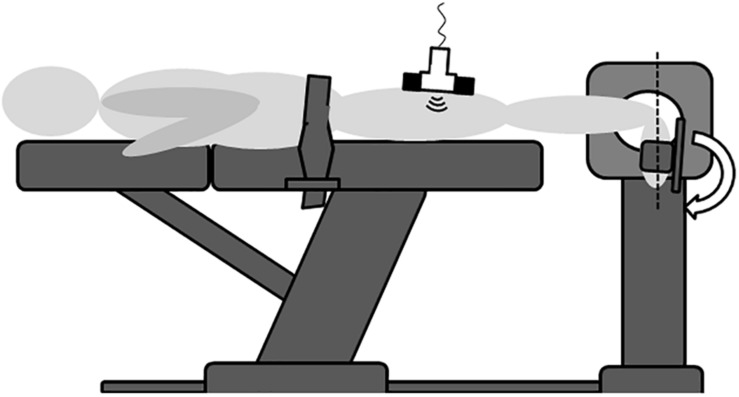
Schematic illustration of the experimental set-up. In the prone position and with the knee extended, the ankle joint was passively moved between plantar flexion and dorsal extension (arrow shows way into dorsal extension). Maximal dorsal extension was calculated as the distance from a calibrated neutral zero position (dashed line). Ultrasound recordings were made over the semimembranosus muscle in order to estimate tissue displacement induced by the ankle motion. EMG (not depicted here) used as biofeedback ensured that no voluntary muscle activity occurred. Pelvic fixation with a strap was required to prevent body motion induced by the dynamometer action. As the belt was not in contact with the Hamstrings, it did not affect tissue displacement.

A schematic depiction of the experimental approach is shown in Figure 1. All experiments were conducted in the same room and at constant temperature and daytime. The participants were positioned in a standardized prone position on an isokinetic dynamometer (Cybex Norm, Cybex, Ronkonkoma, New York, United States), having their ankle joint axis aligned with the rotational axis of the device. A fixation belt was attached over dorsal pelvis (thus not compressing the Hamstring muscles) to prevent body movement. In the experiment, the ankle (tested leg chosen randomly) was moved passively between plantar flexion and maximal achievable dorsal extension by means of the dynamometer’s continuous passive motion function. During the measurements, ankle joint angle [°], relative to the neutral zero position, was constantly recorded by the device. Three repetitions, averaged for analysis, were performed at an angular velocity of 5°/s (Krause et al., 2019). The participants were instructed to remain completely passive, avoiding any voluntary muscle activity. To confirm this, surface electromyography (Biopac MP 160, Biopac Systems Inc., Goleta, CA, United States) was used to monitor muscle activity, providing the participants with live biofeedback. After preparation of the skin (shaving and alcohol cleansing), Ag-AgCl electrodes (8 mm diameter) were positioned over the muscle bellies of the m. semimembranosus, m. gastrocnemius, m. quadriceps femoris and m. tibialis anterior as well as over the lateral malleolus and the lateral femur condyle with the latter two being reference electrodes. Sensor placement was determined according to the SENIAM (surface EMG for non-invasive assessment of muscles) recommendations (Hermens et al., 2000). Electrodes were placed at 1/3 of the distance between the ischial tuberosity and the knee’s medial joint line (semimembranosus), on the most prominent bulge of the gastrocnemius muscle, at 50% of the distance between the anterior spina iliaca superior and the superior part of the patella (quadriceps femoris) and at 1/3 of the way between the tips of the fibula and the medial malleolus (tibialis anterior). Data were sampled at a rate of 1.600 kHz and filtered with a high- and low-pass filter of 10 and 500 Hz, respectively.

In order to familiarize the participants with the device and measurement conditions and to practice keeping the lower leg muscles inactive, a warm-up of three flexion-extension cycles was performed prior to the actual measurements. This approach has already been used successfully in a previous, similar study (Krause et al., 2019).

A high-resolution ultrasonography device (My Lab 70, Esaote Biomedica, Genoa, Italy) was used to assess tissue displacement upon ankle joint movement. Video recordings, depicting the soft tissue of the dorsal thigh, were made with a linear array transducer (custom-made, 100 mm × 8 mm, 7.5 Hz) positioned over the belly of the semimembranosus muscle. To prevent artifacts induced by variations in pressure to the skin, a custom made template consisting of thermoplastic polymer was used for fixation (Cruz-Montecinos et al., 2015). To detect potential probe movement over the skin, acoustically reflective markers (thin stripes of micropore tape) which are clearly visible in the ultrasound image, were placed on the skin (Morse et al., 2008).

### Outcome

To reveal the spatial relations in the area of interest of the United States image, the thickness of the subcutaneous tissue and the deep fascia of the semimembranosus muscle were determined using ImageJ (NIH, Bethesda, MD, United States). Five equidistant measurements were taken at rest and averaged.

**FIGURE 2:**
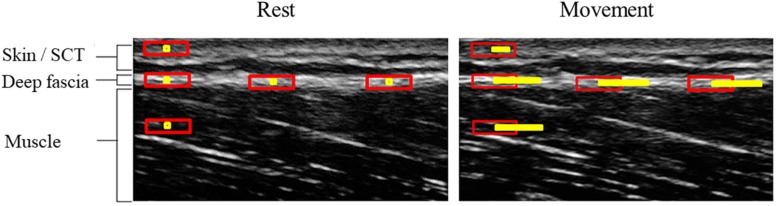
Exemplary visualization of ultrasound-based cross-correlation analysis. Equidistant ROIs (red rectangles) are selected at rest (*left image*). In this example, five ROIs have been positioned: three within the fascia and one in each, the subcutaneous tissue (SCT) and the muscle. Upon movement (*right image*), pixel displacements relative to the center of the non-moving ROIs (small yellow square inside the red rectangles in left picture) are tracked (yellow line in right picture). The end of the line indicates the maximal displacement, which is computed by the software algorithm.

The maximal horizontal displacement of the semimembranosus muscle [mm distance from resting position] during maximal passive ankle dorsal extension represented the primary outcome. It was quantified using a frame-by-frame cross-correlation analysis of the obtained ultrasound videos, which reveals the maximum displacement of the muscle relative to the zero position without ankle movement (see Figure 2). The employed algorithm, created in MATLAB (The MathWorks, Inc., Natick, MA, United States), was developed by Dilley et al. (2001) and has been shown to represent a highly reliable method to quantify tissue displacement (ICC: 0.77 to 0.99). Briefly, the software calculates the correlation coefficient between the pixel gray levels of successive frames within previously defined, rectangle-shaped regions of interest (ROI) of the successive frames. The pixel shift revealing the highest coefficient represents the relative movement between two frames. In video recorded in this trial, six equidistant ROIs (approximate size: 5 × 1 mm) were placed within the semimembranosus. Mean maximal horizontal displacement of the ROIs was calculated and analyzed as quantification of muscle displacement. Excellent reliability of this approach (use of the described software and six ROIs including interpretation procedure) has been demonstrated in a previous trial of our workgroup, which also examined fascial displacement in the thigh (Krause et al., 2019).

### Data Processing and Statistics

The recorded ultrasound, dynamometer and EMG data were synchronized using a common electrical impulse delivered through an external trigger to the interface of the employed software (Acqknowledge, Biopac Systems Inc., Goleta, CA, United States). Besides providing biofeedback, EMG data were used to detect involuntary muscle activity with two approaches implemented in the software package: The first was based on a normalization against maximum voluntary contraction (MVC), which was determined by means of isometric contractions of the included muscles. Here, muscle activity was defined as any root mean square values (0.03 s) >5% MVC. The second was based on the algorithm of Hodges and Bui (1996). Briefly, it determines the mean and standard deviation of the signal during a period of 0.25 s under resting conditions and creates a filtered average rectified value (ARV). It then extracts the variance with regard to the noise by means of dividing the difference between the ARV and the mean by the standard deviation. For the resulting signal, the median is calculated for the entire waveform. Any activity exceeding this median for at least 0.1 s is considered as muscle activity. Once one of the two methods described detected muscle activity, the corresponding trials were discarded from analysis.

Regarding the kinematic data indicating maximal ankle dorsal extension (°) and horizontal tissue displacement (mm), mean values were calculated for the three movement cycles in each condition. To answer the question as to whether ankle movement leads to cranial displacement of the semimembranosus myofascial soft tissue, a twofold approach was chosen. Firstly, the one-sample *t* test and 95% confidence intervals (95% CI) were used in order to examine whether movement of the semimembranosus occurred (systematic difference to zero). Potential discrepancies between men and women were detected using the *t* test for independent samples. Effect sizes of both tests were calculated and interpreted according to Cohen (1992) as small (*d* = 0.2), medium (0.5) or large (0.8). Secondly, to identify significant associations between ankle dorsal extension and semimembranosus displacement, Spearman’s rank correlation was used. According to Evans (1996), resulting coefficients were graded as poor (<0.2), weak (0.2 to 0.4), moderate (0.4 to 0.6), strong (0.6 to 0.8) or optimal (>0.8). Finally, to demonstrate the reliability of the software algorithm for tissue displacement, the intraclass correlation coefficient was used (Bland and Altman, 1996). Based on the suggestions of Fleiss (1986), resulting reliability values were classified as poor (<0.4), moderate (0.4 to 0.75), or excellent (>0.75). All calculations were made with BiAS for Windows 11.2 (Goethe-University, Germany); the significance level was set to α = 0.05.

## Results

All participants completed the experiment. However, while the analyses of the EMG confirmed absence of muscle activity in 10 individuals, one showed above-threshold values and hence, the corresponding data were not included in the inferential statistics. The subcutaneous tissue, per average, had a thickness of 3.80 ± 2.17 mm. With 1.32 ± 0.49 mm, the deep fascia was about two thirds smaller.

### Hamstring Displacement

Calculating tissue displacement was highly reliable, revealing almost perfect agreement between the five repetitions (ICC = 0.81, 95%CI: 0.66 to 0.97, *p* < 0.0001). Corrected for probe movement over the skin, which was negligible (0.09 ± 0.10 mm), passive ankle dorsal extension induced a significant caudal semimembranosus displacement of 5.76 ± 2.67 mm (95%CI: 3.86 to 7.68, *p* < 0.0001, *d* = 2.16). The highest value (10.87 ± 1.57 mm) was registered in a 22-year old female while the lowest displacement was found in a 24-year old male (1.22 ± 0.64 mm). In sum, higher values (6.74 ± 3.03 mm) occurred in women when compared to men (5.12 ± 2.46 mm), however, despite a moderate effect size (*d* = 0.60), this difference did not reach statistical significance (*p* = 0.38).

### Correlation With Ankle Dorsal Extension

**FIGURE 3:**
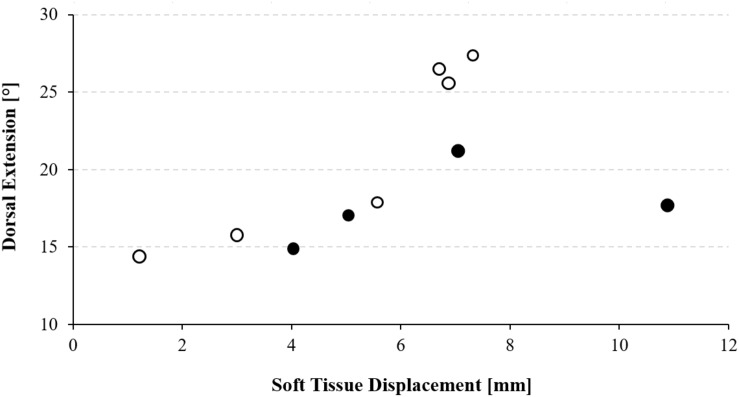
Scatter plot showing the relation between maximal ankle dorsal extension and dorsal thigh soft tissue displacement. Black circles represent males, open circles represent females.

Maximal ankle dorsal extension, per average, was 19.8 ± 5.0 degrees. Spearman analysis revealed a strong positive correlation between the extent of caudal semimembranosus displacement and dorsal extension (*p* = 0.02, rho = 0.76), which suggests that higher ankle movement amplitudes were associated with larger displacements of the semimembranosus muscle (Figure 3).

## Discussion

The mechanical role of the soft tissue has been a recent focus of fascia research (Zügel et al., 2018). However, despite compelling evidence from cadaver experiments, pointing toward the occurrence of in-series force transmission effects across myofascial continuities (Krause et al., 2016), there has been a lack of *in vivo* studies regarding this topic, hitherto (Zügel et al., 2018). Our trial shows that maximal ankle dorsal extension is associated with significant caudal displacements of the semimembranosus muscle and its encapsulating fascia. This finding may explain the remote exercise effects detected in previous studies: It had been shown that stretching or self-myofascial release treatments induced flexibility increases in neighboring or even more distant cranial joints (Grieve et al., 2015; Wilke et al., 2016b, 2017, 2019a; Joshi et al., 2018). Although representing an intriguing observation, the occurrence of non-local changes in such functional outcome could not only be related to a force transmission across myofascial continuity but also be due to other factors such as systemic neural adaptations, i.e., altered stretch tolerance. Against this background, the remote soft tissue displacements established here may, in fact, demonstrate that mechanically relevant amounts of force are transferred to serially connected skeletal muscles in the living organism.

As indicated, this is one of the first studies examining serial myofascial force transmission under *in vivo* conditions. The only similar trial conducted by Cruz-Montecinos et al. (2015) demonstrated a coupling of pelvic movement and displacement of the gastrocnemius fascia. The absolute magnitude of fascial displacement was higher in our data (5.8 mm vs. 1.5 mm). This difference may be explained by various factors, including differences in the imaged muscle (semimembranosus vs. gastrocnemius), the moved joint (ankle vs. pelvis) and the direction of force transmission (caudal-cranial vs. cranial-caudal). Notwithstanding, a common finding of both studies was the strong correlation of local ROM alterations and consecutive remote tissue displacements.

The practical implications of our research span from sports performance to musculoskeletal disorders. Coaches and exercise professionals should be aware that stretching treatments do affect both the targeted tissue but also morphologically linked skeletal muscles. The relevance of the exercise position, for instance having the knee extended or not when targeting the calf, thus extends beyond the question of mono- or bi-articularity of muscles. In essence, it may be argued that fascial tissues represent a potential contributor to restrictions in flexibility, which would be in accordance with previous data (Wilke et al., 2018). Besides its potential relevance under normal conditions, myofascial force transmission could also play a role in the development of overuse disorders. It had been speculated that the occurrence of non-local abnormalities (e.g., increased hamstring stiffness in patients with plantar fasciitis) stems from a pathologically altered/excessive degree of myofascial force transmission (Wilke et al., 2019a). In order to further substantiate this assumption, it seems of interest to conduct similar experiments in both healthy individuals and patients with musculoskeletal disorders.

When interpreting the novel findings, some noteworthy methodological considerations need to be made. Firstly, the measured soft tissue displacement represents a highly plausible surrogate but not a direct measure of transferred force. While this represents a rather theoretical concern, the effects observed must not only stem from a serial force transmission. Ankle movement has been demonstrated to modify the stiffness of the sciatic nerve. As it crosses the knee joint, it may have an impact on the mechanics of the thigh (Andrade et al., 2016). In addition, the fascial bands spanning from the gastrocnemius muscle to the dorsal thigh do not only attach to the semimembranosus but also to the other parts of the Hamstrings. Previous research revealed substantial mutual interaction between muscles located in parallel (Huijing and Baan, 2001). If force was hence transmitted from the calf it is tenable to assume that it also reached the biceps femoris and the semitendinosus. As a consequence, particularly the forces acting on the latter (pulling it in the caudal direction in the same way as occurring with the semimembranosus), could have induced a (minor) part of the muscle displacement. Another issue relates to the sample size of our investigation. Evidently, it was sufficient to reveal the presence of semimembranosus motion and its correlation to ankle movement. However, our study may have been underpowered with regard to the influence of sex: female participants had higher displacements but the significance threshold was failed for this observation. Future studies, further delineating the role of sex on serial force transmission are therefore warranted. Finally, a couple of additional outcomes may be of particular value for upcoming research. Besides visualizing other muscles (e.g., those located in parallel and the calf muscles), it would be intriguing to measure the pennation angle in order to complement the displacement data. From a mechanical point of view, our experimental set-up did not allow for a judgment of tissue stiffness which could be another relevant effect modifier. It may be argued that higher stiffness of the implicated transmitting structures (in this case the gastrocnemius) would allow for a higher degree of force transmission. One possibility to assess this outcome would be using the isokinetic dynamometer to calculate the passive resistive torque produced during ankle movement. As a supplement, elastography, which has recently been used to study the mechanical properties of the lower leg muscles (Ates et al., 2017), could be added to gain further insight the mechanical properties of the leg muscles. Particularly, it would allow the measurement of stiffness changes in the entire Hamstring group in order to quantify the degree of myofascial force transmission from muscle arranged in parallel.

## Conclusion

Our study demonstrated under *in vivo* conditions that mechanical force is transmitted between the ankle and the dorsal thigh upon passive stretching of the gastrocnemius. This finding may represent the morphological substrate of remote exercise effects occurring after treatments based on myofascial chains. The correlation of semimembranosus displacement and ankle dorsal extension, furthermore, may suggest that fascial tissue can restrict flexibility. Future research should elucidate the functional implications of the observed transmission effects in sports and disease.

## Data Availability Statement

The datasets generated for this study are available on request to the corresponding author.

## Ethics Statement

The studies involving human participants were reviewed and approved by the University Research Ethics Committee of Liverpool John Moores University. The patients/participants provided their written informed consent to participate in this study.

## Author Contributions

JW contributed to the concept, design, data acquisition, analysis and interpretation, drafting and critical revision of the manuscript, and final approval of the manuscript. HD contributed to the data acquisition, critical revision of the manuscript, and final approval of the manuscript. ST contributed to the analysis and interpretation, critical revision of the manuscript, and final approval of the manuscript. AD contributed to the analysis and interpretation, and final approval of the manuscript. CM contributed to the concept, design, critical revision of the manuscript, and final approval of the manuscript.

## Conflict of Interest

The authors declare that the research was conducted in the absence of any commercial or financial relationships that could be construed as a potential conflict of interest.
